# Acinetobacter Baumannii Native Valve Infective Endocarditis: A Case Report

**DOI:** 10.7759/cureus.11527

**Published:** 2020-11-17

**Authors:** Ismahane Lahmidi, Darar Charmake, Noha Elouafi, Zakaria Bazid

**Affiliations:** 1 Cardiology, Mohammed I University/Mohammed VI University Hospital, Epidemiological Laboratory of Clinical Research and Public Health, Oujda, MAR; 2 Cardiology, Mohammed I University/Mohammed VI University Hospital, Oujda, MAR

**Keywords:** infective endocarditis, nosocomial infections, acinetobacter baumannii

## Abstract

Infective endocarditis caused by Acinetobacter (A.) baumannii is a rare but severe complication that affects seriously ill, hospitalized patients undergoing invasive procedures. It is associated with an increased mortality rate than that of endocarditis due to the HACEK group (Haemophilus species, Aggregatibacter actinomycetemcomitans, Cardiobacterium hominis, Eikenella corrodens, Kingella kingae) gram-negative bacteria. We report a case of a 54-year-old woman who was diagnosed with infective endocarditis caused by A. baumannii three days following her admission to the intensive unit care (ICU). The diagnosis was made on the basis of repeated blood cultures and transthoracic echocardiography, which revealed mobile vegetation attached to the mitral valve. In spite of aggressive therapeutic regimens, outcomes were poor and the patient died. This report underlines the severe nature of A. baumannii infections, which are still associated with a prolonged hospital stay, and increased morbidity, mortality, and medical costs.

## Introduction

Infective endocarditis (IE) is a life-threatening infection involving the endocardial surface of the heart. Although uncommon, IE has a high mortality rate, even with appropriate antibiotic therapy and surgical intervention. Gram-positive cocci, mostly Staphylococcus aureus and Streptococci, are the main causes of infective endocarditis. Gram-negative bacteria and fungi are not frequent pathogens of infective endocarditis [1-2]. Acinetobacter (A.) species are ubiquitous gram-negative coccobacilli and usually cause nosocomial infections, principally ventilator-associated pneumonia and catheter-associated bacteremia, as well as soft tissue and urinary tract infections. Community-acquired infections by Acinetobacter baumannii are increasingly reported [3]. A rare case of native mitral valve endocarditis secondary to A. baumannii is described in the present report.

## Case presentation

**Figure 1:**
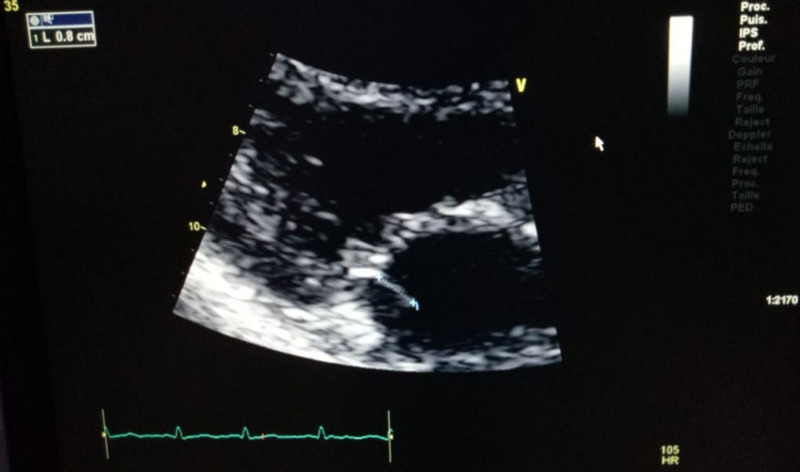
Transthoracic echocardiography showed 0.8 × 0.2 cm vegetation on the patient’s mitral valve in the parasternal long-axis view

**Figure 2:**
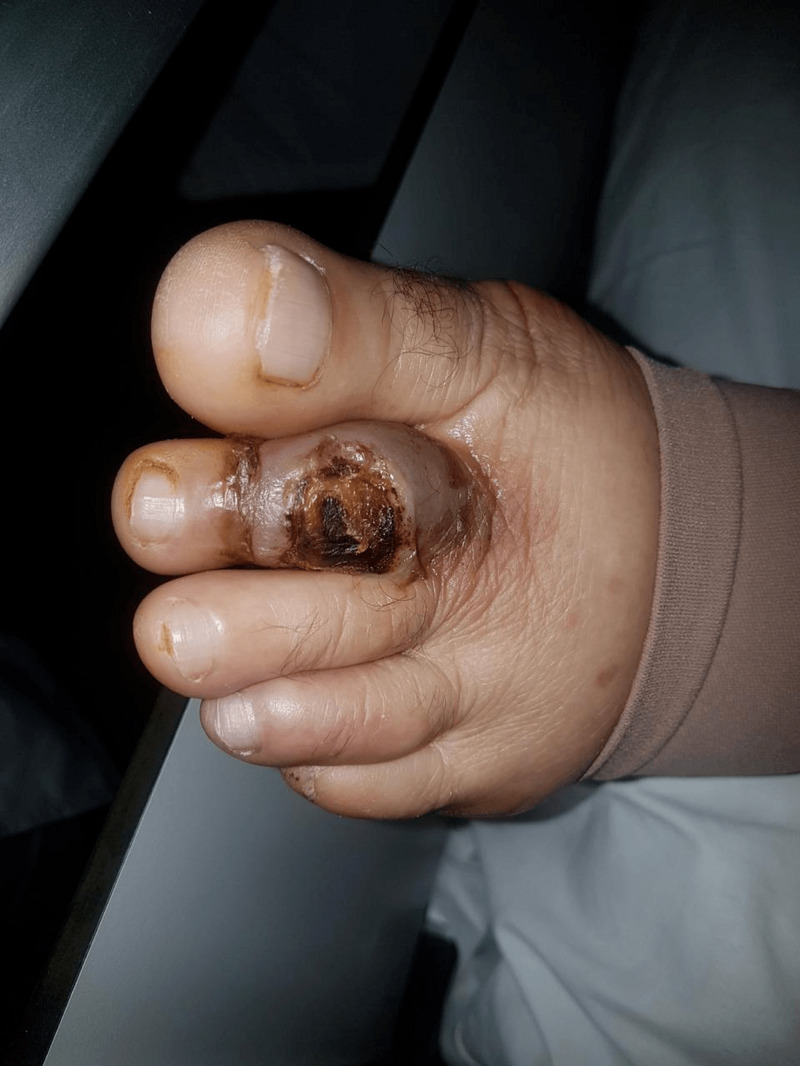
Necrotic ulcer of the patient’s second left toe

A 54-year-old woman presented with a month-long history of painful necrotic ulcer in the left second toe, resistant to conventional treatment. She developed severe sepsis requiring admission to the intensive care unit. On her third day of hospitalization. she developed a high fever of 39.5°C. A new mitral systolic murmur (3/6 intensity) was noted on auscultation. Because of the high suspicion of infective endocarditis, transthoracic echocardiography (TTE) was carried out, which showed mobile vegetation measuring 0.2 × 0.8 cm attached to the mitral valve (Figure 1). A diagnosis of native mitral valve infective endocarditis was established according to the Duke criteria [4]. The patient subsequently was transferred to the cardiac care unit after hemodynamic stabilization. Upon arrival in our unit (cardiac care unit), the patient’s clinical examination revealed a temperature of 39.8°C, blood pressure (BP) of 110/60 mmHg, sinus tachycardia with a pulse of 100/min, and respiratory rate of 20/minute, with an oxygen saturation of 98% on room air. Cardiovascular examination revealed no signs of heart failure, a mitral systolic murmur was heard, and no peripheral stigmata of infective endocarditis were observed. There was a black ischemic ulcer on the dorsum of the left second toe, with oedema (Figure 2). Foul-smelling purulent drainage was noted on her toe ulcer. A dermatology consult was requested for skin ulcer biopsy and culture, a skin punch biopsy of her left second toe ulcer was obtained. Cultures of these skin biopsies grew Staphylococcus aureus and Candida parapsilosis but failed to isolate Acinetobacter baumannii. Histopathology of his skin biopsy reported leukocytoclastic vasculitis. Three blood cultures were carried out at an interval of an hour, as she had a high temperature (40°C); another blood sample was also collected. Two sets of blood cultures yielded Acinetobacter baumannii. The antibiotic susceptibilities of the A. baumannii isolated from our patient are summarized in Table 1.

**Table 1:** Antibiotic susceptibility profile of Acinetobacter baumannii isolated from our patient

Antibiotic	Susceptibility
Piperacillin	Resistant
Piperacillin-tazobactam	Resistant
Ticarcillin	Resistant
Ticarcillin-clavulanate	Resistant
Cefotaxime	Resistant
Ceftriaxone	Resistant
Ceftazidime	Resistant
Imipenem	Resistant
Ciprofloxacin	Resistant
Levofloxacin	Resistant
Amikacin	Susceptible
Gentamicin	Resistant
Tobramycin	Resistant
Tetracycline	Intermediate
Doxycycline	Susceptible
Trimethoprim/sulfamethoxazole	Susceptible

Laboratory test results revealed an elevated C-reactive protein (CRP) (100 mg/L) and erythrocyte sedimentation rate (ESR) (110 mm/hour). We also noted leukocytosis (white blood cell count 22,000 cells/mm^3^, 80% neutrophils) and normochromic normocytic anaemia. Liver function, serum creatinine levels, and the extracted urine sample were normal. Initially, vancomycin, gentamycin, and fluconazole were started but intermittent fever and chills persisted. After blood culture results, polymyxin E was set for 45 days without clinical improvement. Abdominopelvic computed tomography (CT) did not show any abnormalities. Antibiotics were shifted to tigecycline, and early surgical intervention was considered. However, the patient was medically not fit for cardiac surgery with multi-organ failure, and she subsequently died.

## Discussion

A. baumannii is a ubiquitous bacterial pathogen that is largely implicated in nosocomial infections. It is a pleomorphic, aerobic gram-negative bacillus, non-motile, and widely distributed in the environment. Its ability to survive even in hostile conditions allows it a wide circulation in the hospital setting. It is often spread to patients from workers’ hands or healthcare devices [5]. There are many risk factors associated with Acinetobacter spp. bacteremia-like invasive procedures, mechanical ventilation, and surgery [6]. Among 17 cases of A. baumannii endocarditis reviewed [7], the inciting events were dental work, intravenous drug abuse, septic abortion, stab wounds, burns, intravenous catheters, and open-heart surgery. None of these events was noted in our patient except for the hemodialysis catheter in his right internal jugular vein. Most likely, in our patient, the infection is secondary to invasive procedures (intravascular catheter). We postulated also that the skin lesions (left second toe ulcer) could be the likely nidus of the infection.

The most frequent sites of infection are urinary and respiratory tracts, bloodstream, and surgical sites. A few cases of A. baumannii endocarditis have previously been reported. Prosthetic valves were involved in the majority of these cases [8-9].

Native valve endocarditis due to A. baumannii is a life-threatening infection, with a worse prognosis than the prosthetic valve form, perhaps because of the poor index of suspicion and the delayed initiation of treatment. Among the described patients in the literature, 16.6% of patients with prosthetic valve endocarditis died vs. 33% of patients with native valve endocarditis [7].

Echocardiography is essential for diagnosis. It should be performed in all patients with a moderate or high suspicion of infective endocarditis to confirm the diagnosis and guide the timely institution of appropriate therapy, including the need for surgical intervention. It also helps in determining prognosis [10]. Transesophageal echocardiography (TEE) is much more sensitive than transthoracic echocardiography (TTE), with increased diagnostic accuracy. In our case, the diagnosis was easily made without resorting to TEE.

It is evident that the outcome of patients with A.baumannii infective endocarditis is poor due to its growing resistance to antimicrobials. Imipenem is usually the first-line antibiotic against infections caused by A. baumannii [7,11], Empirical broad-spectrum therapy (e.g., imipenem/cilastatin) for Acinetobacter infections should be considered in any patient with infective endocarditis when gram-negative coccobacilli are isolated from blood cultures [7]. The present isolate in our patient was resistant to imipenem.

No surgical intervention was reported in a study of native-valve endocarditis due to Acinetobacter species [7], and a survival rate of 65% was reported. Our patient met indications for surgical treatment [12], but she was at high risk considering her poor general condition.

## Conclusions

This report concluded that native valve infective endocarditis caused by A. baumannii is rare. The relevance of this case is centred on the fact that infective endocarditis due to the dreaded A. baumannii is not a common cause but a possible cause of death in patients who have stayed in the intensive care unit; hence, the necessity of nosocomial infection control and prevention measures.
